# Examining shifts in medical students’ microanalytic motivation beliefs and regulatory processes during a diagnostic reasoning task

**DOI:** 10.1007/s10459-014-9549-x

**Published:** 2014-09-11

**Authors:** Timothy J. Cleary, Ting Dong, Anthony R. Artino

**Affiliations:** 1 Rutgers, The State University of New Jersey, New Brunswick, NJ USA; 2 Department of Medicine, Uniformed Services University of the Health Science, Bethesda, MD USA; 3 Graduate School of Applied and Professional Psychology, Rutgers, The State University of New Jersey, 152 Frelinghuysen Road, Piscataway, NJ 08854-8085 USA

**Keywords:** Diagnostic reasoning, Feedback, Microanalytic assessment, Motivation, Self-efficacy, Self-regulated learning

## Abstract

This study examined within-group shifts in the motivation beliefs and regulatory processes of second-year medical students as they engaged in a diagnostic reasoning activity. Using a contextualized assessment methodology called self-regulated learning microanalysis, the authors found that the 71 medical student participants showed statistically significant and relatively robust declines in their self-efficacy beliefs and strategic regulatory processes following negative feedback about their performance on the diagnostic reasoning task. Descriptive statistics revealed that changes in strategic thinking following negative corrective feedback were most characterized by shifts away from task-specific processes (e.g., integration, differentiating diagnoses) to non-task related factors. Implications and areas for future research are presented and discussed.

## Introduction

Self-regulated learning (SRL) has been described as a process through which individuals exert control over their behaviors, cognitions or affect, and the environment as they engage in goal-directed activities (Boekaerts et al. 2000). In addition to the various theoretical perspectives of SRL in educational psychology, medical education researchers have studied several related constructs, such as self-directed learning, reflective practice, and self-assessment (see Cleary et al. 2013 for an overview). Despite the diversity of constructs, most researchers would agree that successful students are those who exhibit regulatory or control characteristics, such as strategically engaging in learning and clinical activities and being flexible and adaptable when faced with learning challenges or obstacles. High achieving individuals are also particularly adept at proactively gathering information about their task performance, either through self-monitoring or through efforts to obtain feedback provided by experts, such as supervisors, faculty, or other instructors. From an SRL perspective, self-generated or externally provided feedback is important because it helps to determine whether and how individuals will reflect on and improve their approach to learning or clinical tasks (Hattie and Timperley 2007; Sargeant et al. 2009).

There is ample evidence across various domains showing that feedback about performance can lead to enhanced outcomes (Archer 2009; Hattie and Timperley 2007; Shute 2008; Wigton et al. 1986). However, two important caveats must be underscored. First, in medical education contexts, researchers have identified a disconnect between student and faculty perceptions regarding the quality and frequency of faculty feedback provided to students (Gil et al. 1984; Libemeran et al. 2005). Although students and faculty perceive feedback to be a critical factor in reducing student errors and enhancing overall performance, faculty members or supervisors generally believe that their feedback is of high quality (i.e., timely, specific, and effective), while trainees are often dissatisfied and highly critical of this feedback (Bing-You et al. 1997; Libemeran et al. 2005).

Second, feedback is an umbrella term that includes various types or levels (Hattie and Timperley 2007; van de Ridder et al. 2008). Although different terminology has been used to describe these varying forms of feedback, there is general agreement that some feedback is more effective than others. For example, feedback that directs students’ attention to personal attributes or qualities of the “self” (e.g., “You are good at doing venepuncture”; “You had a tough time on this activity”) or feedback that only conveys information about correctness on a task (e.g., “Your diagnosis is incorrect”) tend to be less effective than feedback explaining *why* an individual may have struggled to perform well, particularly in relation to the strategy or process that he or she used to perform a given task (e.g., “You did a good job of planning how to approach this case” or “You did not integrate all of the identified symptoms before making a diagnosis”; Archer 2009; Hattie and Timperley 2007; Shute 2008).

From an SRL perspective, when feedback is restricted to simple forms of corrective or task performance information (e.g., correct/incorrect), students will be less likely to engage in error analysis or to re-strategize how they approach the task and may even disengage and/or withdraw (Hattie and Timperley 2007; Zimmerman 2000). However, to our knowledge there have been very few attempts in the medical education literature to document and illustrate how the regulatory responses of medical trainees may shift and change during specific tasks following corrective feedback. Thus, the primary purpose of the current study was to address this gap in the literature by targeting shifts in medical students’ strategic regulatory processes and motivation beliefs during two iterations of a specific case-based diagnostic reasoning scenario.

## Theoretical and conceptual framework

**Fig. 1:**
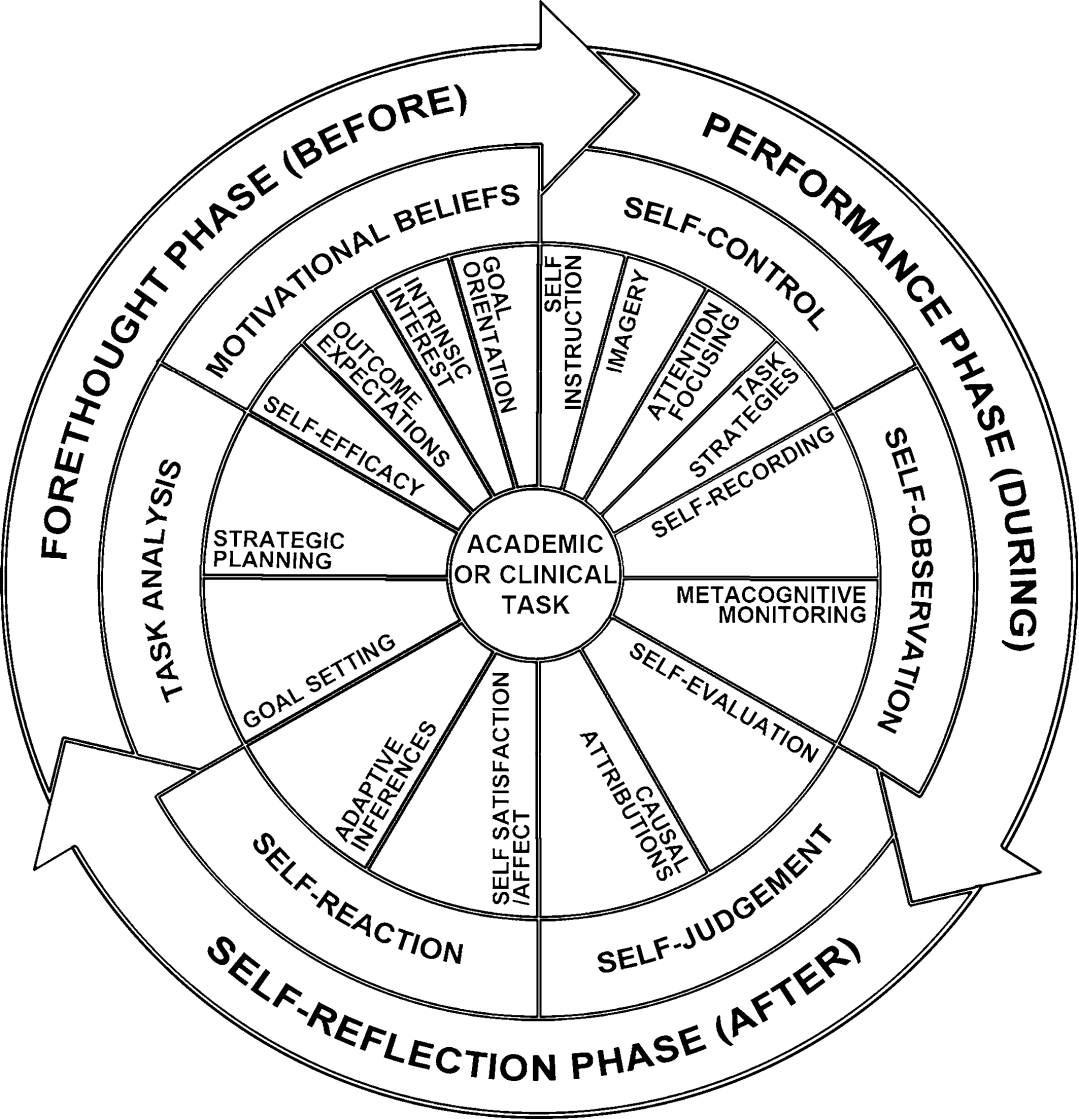
A three-phase, cyclical model of self-regulated learning (SRL). The model presented here is adapted from Zimmerman (2000) and depicts three sequential phases of SRL: forethought (*before*), performance (*during*), and self-reflection (*after*). The model also shows, within each phase, the sub-processes of SRL. Adapted, with permission, from Artino and Jones (2013)

We used a social-cognitive model of SRL as the theoretical framework for the current study (Bandura 1986; Zimmerman 2000). From this perspective, SRL is conceptualized as a multi-dimensional process involving a set of thoughts, feelings, and actions that are intentionally and proactively used by learners to attain personal goals (Zimmerman 2000). The process of SRL is depicted in terms of a three-phase cyclical feedback loop (see Fig. 1), involving forethought (before the task), performance (during the task), and self-reflection (after the task). Forethought phase processes, which include establishing *personal goals* and *strategic plans* as well as motivation beliefs, such as *self*-*efficacy*, occur prior to beginning a learning or performance activity. Goal-setting involves deciding upon specific outcomes of learning or performance while strategic planning refers to the selection of specific strategies that will facilitate the acquisition or display of skill (Zimmerman 2000). Finally, self-efficacy has been defined as a contextualized belief about one’s personal capabilities to execute specific behaviors at designated levels of performance on a specific activity (Bandura 1997).

In the performance phase of the feedback loop, which occurs during a learning task or activity, highly self-regulated individuals will often employ self-control tactics to manage their behaviors, affect, and cognition (e.g., attention-focusing, self-instruction statements) and will proactively attempt to keep track of their learning progress as well as the types of actions and thoughts they exhibit, a process called *metacognitive monitoring*. Thus, it is during the performance phase when individuals will implement their strategic plan and will attempt to gather information about aspects of their task performance that may need refinement. The feedback that students gather during this phase can either be self-generated, such as when students record the specific concepts that are confusing when reading a textbook, or externally provided, such as when a faculty member conveys to students whether they produced an accurate clinical diagnosis (Cleary et al. 2012).

The final phase of this feedback loop, called self-reflection, occurs after individuals engage in a learning activity. Using the self-generated or externally provided feedback as data sources, highly self-regulated individuals will evaluate whether their performance matched their personal goals (*self*-*evaluation*), will identify the reasons for this level of performance (*attributions*), and will draw conclusions about how to adapt prior to future learning attempts (*adaptive inferences*). An iteration of the cyclical feedback loop is completed when self-reflection phase processes impact students’ forethought processes prior to a subsequent learning or performance activity.

## Purpose of study

Using the three-phase cyclical feedback loop as our conceptual framework, we used inferential and descriptive statistical procedures to explore how second-year medical students responded to corrective feedback (i.e., correct or incorrect diagnosis) during a specific diagnostic reasoning task. We examined two specific research questions. First, do second-year medical students, who can be considered novice diagnosticians, show significant declines in their self-efficacy and strategic thinking as they engage in multiple iterations of a challenging clinical reasoning task? Although we expected to observe decreases in these processes, we did not make any a priori predictions regarding the size of the changes or whether such changes would occur after the first or second iteration of the task. Second, if changes in students’ strategic thinking are observed, what are the primary types of responses that students begin to exhibit following the task feedback?

Addressing these two objectives was important given the paucity of research examining how feedback impacts regulatory processes in real time as well as the lack of empirical data documenting that shifts in strategic thinking correspond to changes in motivation beliefs, such as self-efficacy. Further, a highly contextualized interview protocol, called SRL microanalysis, was utilized to assess SRL processes in this study because of its' utility in capturing the cyclical-phase SRL processes exhibited by individuals as they engage in specific learning or performance activities (Cleary et al. 2012; Cleary and Sandars 2011; DiBenedetto and Zimmerman 2013).

## Method

### Participants and study context

Three hundred and forty-two second-year medical students were invited to complete the study, with 71 students (21 %) volunteering to participate. All students were recruited from an Introduction to Clinical Reasoning (ICR) course offered at the F. Edward Hébert School of Medicine, Uniformed Services University of the Health Sciences (USU). The participating sample included 46 men (65 %), which is similar to the overall medical student population at USU (71 % men). At the time of the study, USU offered a traditional 4-year curriculum: 2 years of basic science courses followed by 2 years of clinical rotations (clerkships). The participants volunteered for the study, but they were awarded three extra credit points in the ICR course for their participation. This study was part of a larger project that was administered across two academic years (2010–2011 and 2011–2012) and was approved by the university’s Institutional Review Board. Prior to participation in the study, all student volunteers provided written informed consent.

### Procedures

The last author individually administered a diagnostic reasoning task to participants during a 25–30 min session outside of the normal ICR class time during the last month of the course. The participants were instructed to read a one-page paper description of the case depicting diabetes mellitus. The case scenario included in the study was identified as a difficult case based on consensus among a group of medical educators at USU who had previously developed and pilot tested the case with medical students during a diagnostic reasoning course. The challenging nature of the case was supported by the fact that all participants failed to provide a correct diagnosis during the current study. Including a difficult case was paramount given our interest in examining shifts in students’ SRL responses to corrective feedback following failure experiences.

**Fig. 2:**
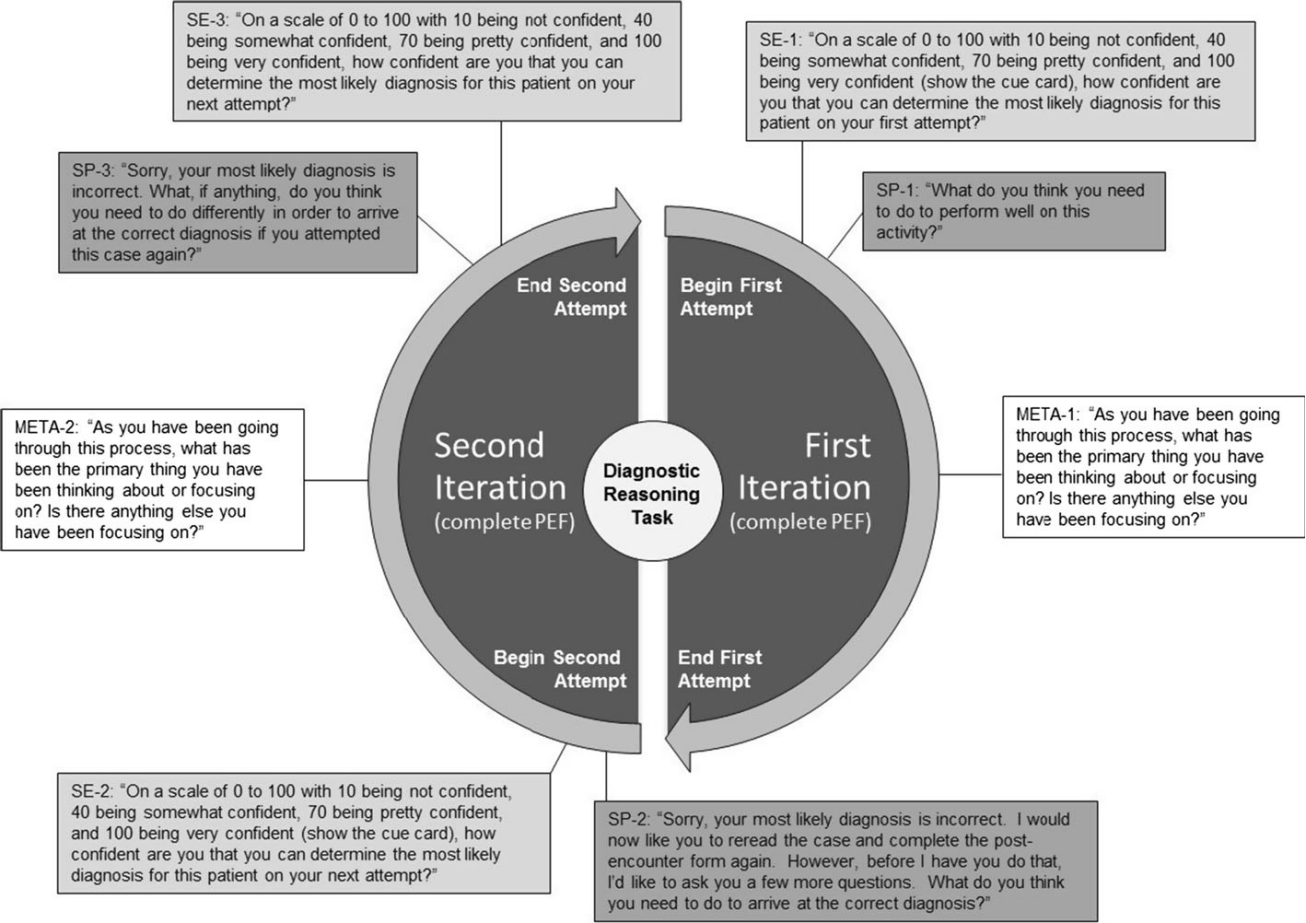
To evaluate shifts in students’ SRL processes and self-efficacy beliefs during the multiple iteration activity, an SRL microanalytic interview was administered to the participants at different points during the task. The form and sequence of each interview question is provided here

All participants were afforded the opportunity to use a post-encounter form (PEF) as a guide to developing and generating an accurate diagnosis. The participants were familiar with the components of the PEF (e.g., summary statement, problem list, differential diagnosis) because they had received training on how to use this form as part of the 10-month ICR course. Following their initial attempt to provide the correct diagnosis, the examiner provided simple corrective feedback: “Sorry, your most likely diagnosis is incorrect.” The participants were then given the opportunity to complete another PEF as they engaged in the second iteration of the same clinical reasoning activity. Following their second attempt at generating a most likely diagnosis, the students were again given a similar corrective feedback statement (see Fig. 2).

To evaluate shifts in students’ self-efficacy beliefs and SRL processes during the multiple iteration activity, an SRL microanalytic interview was administered to the participants at different points during the task (see Fig. 2). As stipulated in guidelines for using microanalytic protocols (Cleary et al. 2012), the microanalytic questions were purposefully administered to parallel the temporal dimensions of the task. For example, given that self-efficacy and strategic planning are forethought phase processes, questions targeting these processes were administered *prior* to each iteration of the task. In total, these two SRL processes were assessed at three points: prior to initiating the task, before beginning the second iteration of the task, and prior to a prospective third iteration. Given that metacognitive monitoring is a performance phase process, this measure was administered *during* both the first and second iterations of the task. All sessions were audio recorded and transcribed to ensure accuracy of participant responses during the SRL microanalytic interview.

### Measures

#### Self-efficacy

Using guidelines outlined by Bandura (2006) for developing task-specific self-efficacy measures, a single-item measure was created to examine the participants’ confidence about their ability to generate the correct diagnosis at three separate times during the clinical reasoning task, as described above. Each self-efficacy item used a Likert-type scale ranging from 0 to 100 that was broken down into ten-point increments (e.g., 0, 10, 20, etc.). See Fig. 2 for the wording of each self-efficacy measure during this process.

#### Strategic planning

A single-item, microanalytic measure was used to assess participant plans for approaching the diagnostic reasoning task (Cleary et al. 2012). Participants were asked, “What do you think you need to do to perform well on this activity?” It should be noted that the wording for the second and third administrations of this measure was modified slightly from the initial measure in order to make it more consistent with the flow of the multiple-iteration activity (see Fig. 2 for a description of the specific measures).

Participant responses to the strategic planning question were coded into one of six categories*: task*-*specific process*, *task*-*general process*, *self*-*control*, *non*-*task strategy*, *do not know/none*, and *other*. The coding scheme was an adaptation of microanalytic coding rubrics used in prior research with psychomotor tasks (Cleary and Zimmerman 2001; Cleary et al. 2012). The *task*-*specific process* category involved responses pertaining to five key strategies typically used for diagnostic reasoning tasks: (a) identifying symptoms (b) identifying contextual factors (e.g., social or environmental factors), (c) prioritizing relevant symptoms, (d) integrating/synthesizing symptoms and other case features, and (e) comparing/contrasting diagnoses. These five strategic processes of clinical reasoning were identified using the clinical reasoning literature and through consensus from three expert clinicians. An example of a response coded for this category was, “To group the symptoms and to see the pattern that emerges.” The *task*-*general process* category involved responses pertaining to a general method or procedure to follow, such as “To do all the right steps to solve the case.” The *self*-*control* category involved responses pertaining to effort, focus, concentration, or other management tactics designed to enhance performance on the task (e.g., “To buckle down and focus on doing the right thing”). The *non*-*task strategy* category involved responses pertaining to some outcome or process that was not helpful for completing the target task or one which was not possible given the constraints of the given task (e.g., “To do more studying about this condition”). Finally, the *do not know/none* category involved responses that explicitly indicated that the student did not have a task strategy (e.g., “Nothing really, just do it”), whereas the *other* response category included any response that did not fit into the above categories. Responses were coded independently by two of the authors, and a percent agreement of 88 % was attained. Disagreements were resolved through discussion among all authors.

#### Metacognitive monitoring

We administered a single-item microanalytic question to examine the extent to which the participants focused on strategic processes during each of the two iterations of the clinical reasoning task. Across both iterations, the participants were stopped after they wrote down their first differential diagnosis on the PEF and asked, “As you have been going through this process, what has been the primary thing you have been thinking about or focusing on?” If the participants provided a response, they were asked, “Is there anything else you have been focusing on?” (see Fig. 2).

Student responses were coded into one of seven categories. Four categories were similar to the strategic planning measure, *task*-*specific process*, *task*-*general process*, *self*-*control*, and *other*. Three additional categories were added to this coding scheme: *outcome*, *perceived ability*, and *task difficulty*. The perceived ability category involved responses pertaining to student perceptions of their ability to perform the task and/or their knowledge related to the task. Examples of responses coded for this category were “I was never that good at diagnosing” and “I have no idea what these terms mean.” The *task difficulty* response category involved responses pertaining to the inherent challenges or difficulty level of the task (e.g., “There is not enough information in this case”). Finally, an *outcome* response pertained to getting the correct diagnosis (e.g., “To get the correct diagnosis on my first attempt”). Using similar coding procedures employed for the strategic planning measure, a percent agreement of 88 % was attained, and disagreements were resolved through discussion among all authors.

### Analysis

Prior to addressing our research questions, we first screened the dataset to check for missing data and examine the normality of the variables. Further, given the relatively modest response rate of 21 %, we examined the representativeness of our sample by comparing the participants to non-participants. To address our research questions, we used SPSS 21.0 to calculate descriptive and inferential statistics. Further, given that the strategic planning and metacognitive monitoring measures used a free-response format, and because participants were permitted to provide multiple, codeable responses to each question, we transformed the categorical responses to a metric scale to facilitate interpretation. The scoring system was designed to capture the strategic quality of the participants’ regulatory processes during the specific task, with greatest weight given to responses that reflected one or more of the five strategic steps identified for the diagnostic reasoning task. This scoring system was an adaptation of a prior scoring scheme and was developed based on theory, prior research, and expert consensus (Cleary et al. in press). The scoring system used in the present study is described in detail elsewhere (Artino et al. 2014).

In terms of the first research question, we used repeated measures ANOVA to examine whether there was a linear trend across three time points for the self-efficacy and strategic planning measure (see Fig. 2). We also conducted two separate pairwise comparisons to examine within-group changes in self-efficacy and strategic planning from Time 1 to Time 2 as well as from Time 2 to Time 3. To adjust for inflation of type I error associated with multiple comparisons, we employed a Bonferroni correction procedure for the two comparisons (i.e., dividing the 0.05 alpha level by the number of comparisons), but employed a more conservative approach of considering all three potential comparisons in the family-wise error rate because of the post hoc nature of the analyses. The adjusted alpha level used to determine statistical significance in these analyses was 0.0167 (i.e., 0.05/3). For the metacognitive monitoring question, only two data points were collected; during the first iteration of the task and during the second iteration. Thus, a simple paired *t* test was used to examine these changes.

To address the second research question, we used descriptive analysis to illustrate the quality or nature of the observed shifts or changes in the strategic planning and metacognitive measures following two instances of the negative corrective feedback.

## Results

Preliminary analysis revealed no missing data for the strategic planning measures. However, three individuals did not provide self-efficacy estimates across each of the three time points, and thus were removed prior to conducting inferential statistics targeting self-efficacy. Preliminary analysis comparing participants and non-participants was mixed. There was no difference between the two groups across gender, undergraduate grade point average (GPA), or Medical College Admission Test (MCAT) verbal reasoning scores. However, the study participants displayed a higher first-year GPA (*M* = 3.29, *SD* = 0.45) than non-participants (*M* = 3.04, *SD* = 0.50), *t*(349) = 3.70, *p* < .001.

### Within-group shifts in self-efficacy

**Fig. 3:**
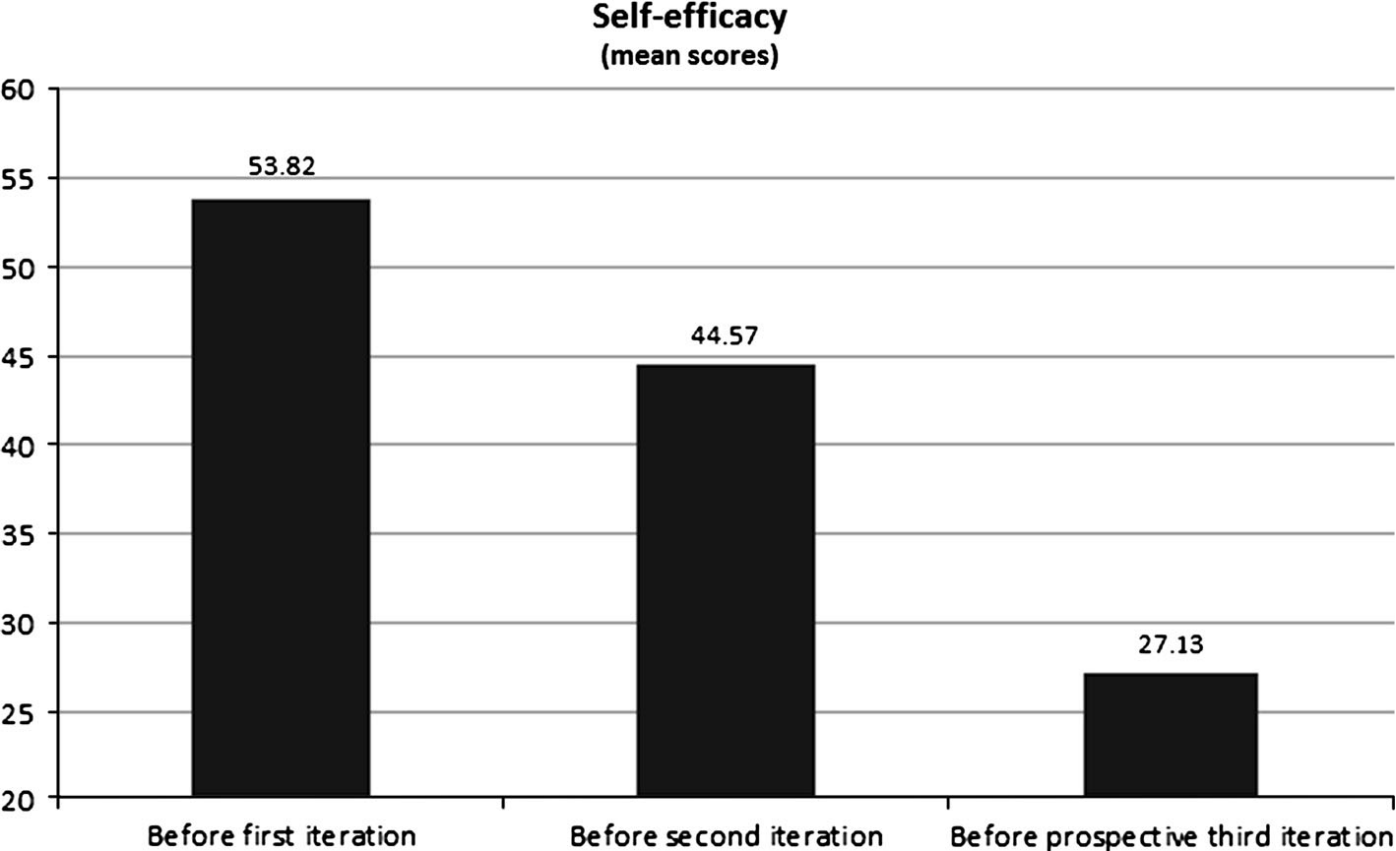
Shifts in mean self-efficacy scores across multiple iterations of the diagnostic reasoning task. All mean values were significantly different from each other at *p* < .001

In terms of self-efficacy, the repeated measure analysis revealed a significant linear trend, *F*(1, 67) = 114.37, *p* < .001, *η*_*p*_ = .631, indicating an overall decrease in students’ self-efficacy during the clinical reasoning task. The size of this effect was quite large (Cohen 1988). In terms of the pairwise comparisons, a statistically significant drop in self-efficacy was observed across both times points: Time 1 to Time 2, *t*(67) = 4.15, *p* < .001, *d* = 0.50; Time 2 to Time 3, *t*(67) = 8.97, *p* < .001, *d* = 0.97. Collectively, these results indicated that the participants showed a medium decrease in their self-confidence after receiving negative corrective feedback following the first iteration of the task, and an even larger decrease following the second round of corrective feedback (see Fig. 3).

### Within-group shifts in strategic regulatory processes

**Fig. 4:**
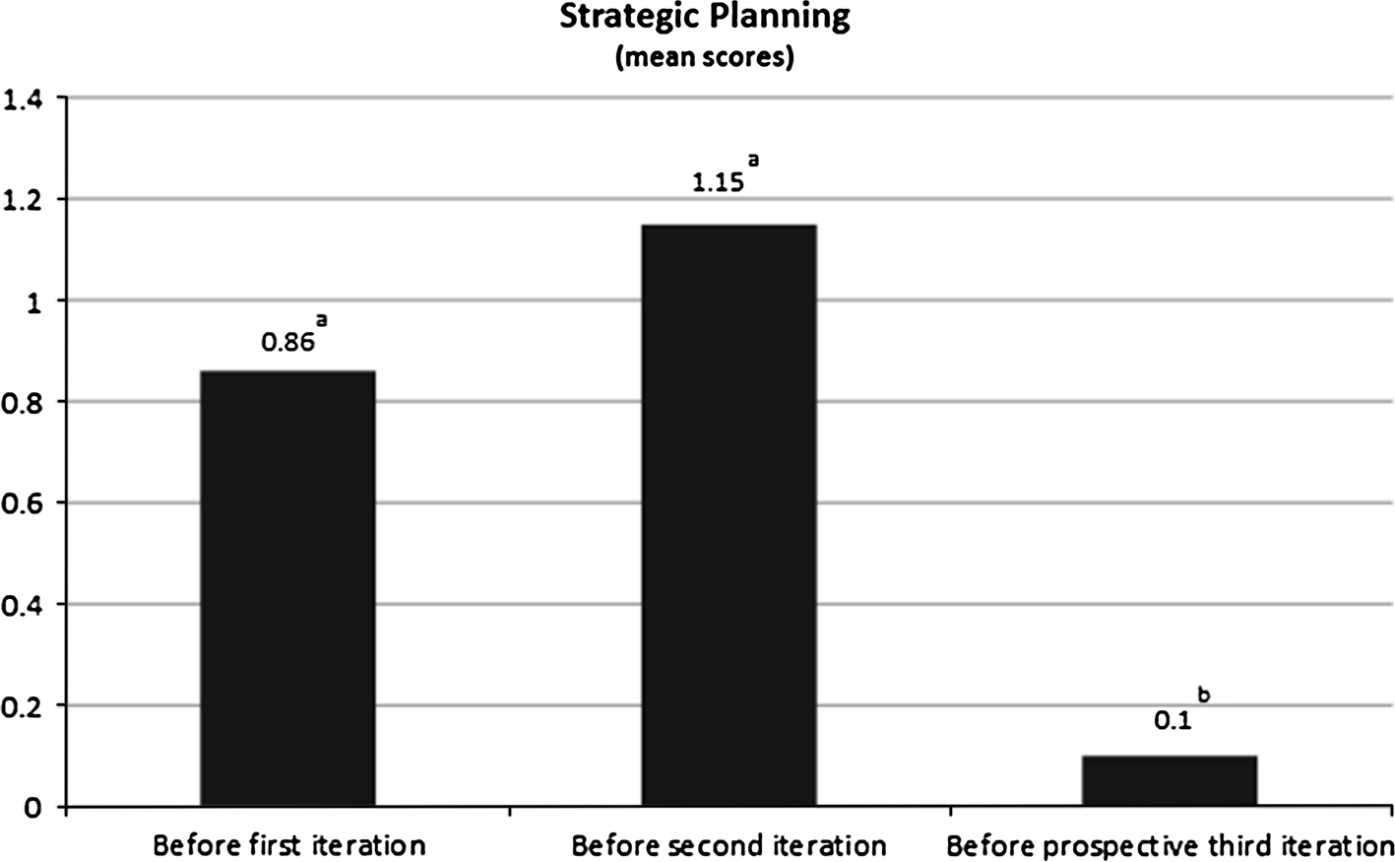
Shifts in mean strategic planning scores across multiple iterations of the diagnostic reasoning task. Bars sharing the same letter did not differ significantly from each other. Statistically significant mean differences were at *p* < .001

Similar to the self-efficacy analyses, we conducted a repeated measures analysis across three time points for the strategic planning measure and then tested two specific pairwise comparisons. Although a statistically significant linear trend was observed, *F*(1, 70) = 5.32, *p* = .024, symbol *η*_*p*_ = .07, a quadratic trend was also found and showed a stronger effect, *F*(1, 70) = 8.54, *p* = .005, *η*_*p*_ = .11. Given that the quadratic trend was relatively robust, it appears that unlike the pattern observed with self-efficacy, there was not a continuous drop in forethought strategic thinking over time during the clinical reasoning task (see Fig. 4). Pair-wise comparisons showed that the change in participants’ strategic plans from Time 1 to Time 2 was not statistically significant, *t*(70) = 1.11, *p* = .269, *d* = .17. However, there was a statistically significant drop in the quality of their strategic thinking from Time 2 to Time 3, *t*(70) = 3.50, *p* = .001, *d* = 0.58. This latter effect was of medium size (Cohen 1988). Altogether, these results suggest that after receiving negative corrective feedback following their first unsuccessful attempt to generate the correct diagnosis, the quality of participants’ strategic thinking did not change. However, after they received similar feedback about their second failed attempt, the participants exhibited a robust decline in their strategic thinking.

Descriptive analyses of participants’ strategic planning responses over time further illustrated this shift away from task-specific strategic thinking (task-specific + task-general processes) to non-strategic information (see Table 1). For example, at Time 1 and Time 2 between 60 and 66 % of the sample provided responses that reflected a focus on the strategic process typically used in diagnostic reasoning. At Time 3, however, only 35 % of the responses referenced these clinical reasoning steps or general process. Of greater importance is that approximately 50 % of the sample at Time 3 either did not develop any plan at all (“I do not know what I would do”) or developed plans that were not possible for the task (e.g., “I need to know more information about this case”). In contrast, at Times 1 and 2, only 17 and 25 % of the sample, respectively, reported these types of ineffective and non-task specific plans.

**Table 1:** Participant responses to the strategic planning microanalytic measure across three time points

Response category	Time 1	Time 2	Time 3
	*n* (%)	*n* (%)	*n* (%)
Task-specific process	24 (33.8)	36 (50.7)	15 (21.1)
Identifying symptoms^a^	12 (50.0)	8 (22.2)	0 (0.0)
Identifying contextual factors^a^	3 (12.5)	6 (16.7)	0 (0.0)
Prioritizing relevant symptoms^a^	2 (8.3)	16 (44.4)	8 (53.3)
Integrating/synthesizing symptoms^a^	11 (45.8)	7 (19.4)	6 (40)
Comparing/contrasting diagnoses^a^	4 (16.7)	8 (22.2)	5 (33.3)
Task-general process	19 (26.8)	11 (15.5)	10 (14.1)
Self-control	11 (15.5)	13 (18.3)	4 (5.6)
Non-task strategies	11 (15.5)	14 (19.7)	26 (36.6)
Do not know/none	1 (1.4)	4 (5.6)	10 (14.1)
Other	18 (25.4)	4 (5.6)	6 (8.5)

Column numbers represent the number (n) and percentage (%) of the total sample of 71 students who provided a particular response category. The total percentage in each column is greater than 100 % because the participants could have provided more than one codeable response to a given question

Time 1 = before first iteration; Time 2 = before second iteration; Time 3 = before prospective third iteration. Time 1 data was presented previously in Artino et al. (2014)

^a^The n’s represent the number of students within the task-specific process category who provided a response coded to one of the five key strategies. The percentage (%) is calculated by dividing the n in a given category by the total number of participants who provided a task-specific response. Thus, for Time 1 the denominator was 24; for Time 2 the denominator was 36 and for Time 3 the denominator was 15

**Fig. 5:**
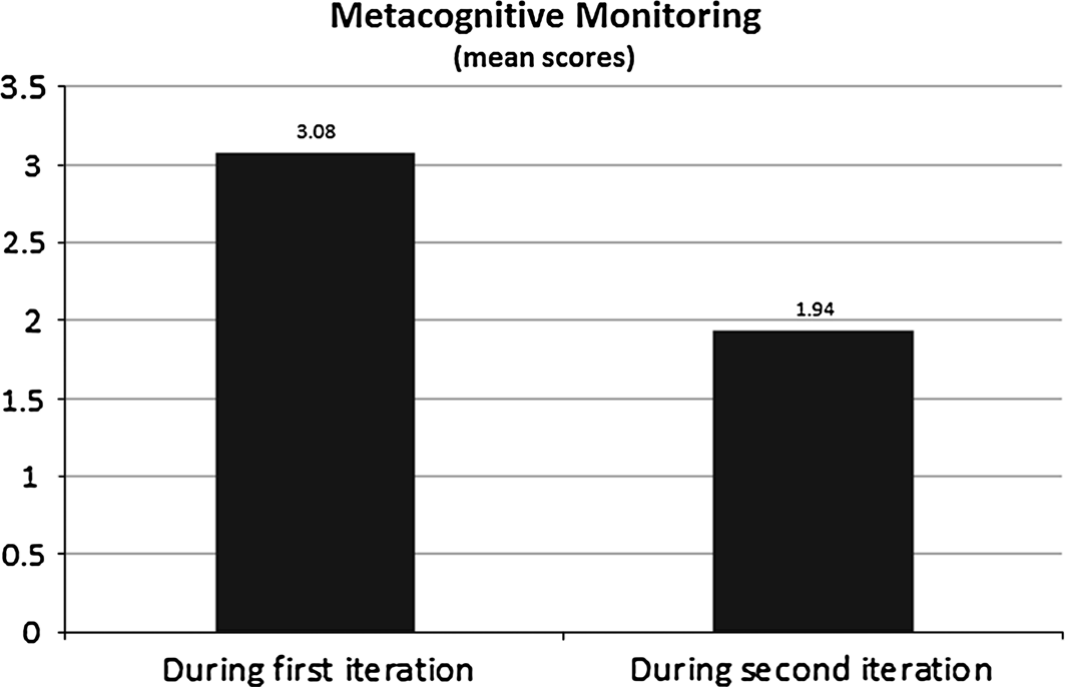
Shifts in mean metacognitive monitoring scores across two time points of the diagnostic reasoning task. The decline in mean values was significantly different at *p* = .005

To evaluate shifts in students’ strategic thinking *during* task engagement, a paired sample *t* test was used to examine differences between the metacognitive monitoring measure administered during the first and second iteration of the task. The results indicated a statistically significant and medium-sized decline in the quality of participants’ strategic thinking from the first to the second iteration of the task, *t*(70) = 2.91, *p* = .005, *d* = 0.49 (see Fig. 5). That is, the participants showed significantly lower levels of task-specific strategic thinking while performing the second iteration of the diagnostic reasoning task than when attempting to provide their initial diagnosis.

Descriptively, although a relatively large percentage of individuals focused primarily on task-specific strategic processes during both iterations of the clinical reasoning activity, relatively fewer individuals focused on this strategic process during the second attempt (see Table 2). Further, approximately 21 % of the sample at Time 2, in contrast to 4 % at Time 1, reported focusing on task difficulty level as the *primary thing* that they were thinking about.

**Table 2:** Participant responses to the metacognitive monitoring microanalytic measure across time points

Response category	Time 1	Time 2
	*n* (%)	*n* (%)
Task-specific process	64 (90.1)	49 (69.0)
Identifying symptoms^a^	33 (51.6)	3 (6.1)
Identifying contextual factors^a^	22 (34.4)	6 (12.2)
Prioritizing relevant symptoms^a^	9 (14.1)	9 (18.4)
Integrating/synthesizing symptoms^a^	38 (59.4)	38 (77.6)
Comparing/contrasting diagnoses^a^	11 (17.2)	13 (26.5)
Task-general process	14 (19.7)	5 (7.0)
Self-control	6 (8.5)	0 (0.0)
Perceived ability	2 (2.8)	1 (1.4)
Task difficulty	3 (4.2)	15 (21.1)
Teacher skill	0 (0.0)	1 (1.4)
Other	5 (7.0)	3 (4.2)

Column numbers represent the number (n) and percentage (%) of the total sample of 71 students who provided a particular response category. The total percentage in each column is greater than 100 % because the participants could have provided more than one codeable response to a given question

Time 1 = during first iteration; Time 2 = during second iteration. Time 1 data was presented previously in Artino et al. (2014)

^a^The n’s represent the number of students within the task-specific process category who provided a response coded to one of the five key strategies. The percentage (%) is calculated by dividing the n in a given category by the total number of participants who provided a task-specific response. Thus, for Time 1 the denominator was 64; for Time 2 the denominator was 49

## Discussion

In this study we examined within-group shifts in medical students’ self-efficacy beliefs and strategic regulatory processes as they attempted to generate an accurate diagnosis of a paper case scenario. This study is important because it provides some insight into the possible mechanisms that go awry when novice learners receive negative corrective feedback. Identifying medical students’ efficacy beliefs as well as the strategic quality of their thinking and actions during clinical tasks has implications for remediation and intervention programs that can help students restructure faulty beliefs and sustain their focus on the key strategic elements of a given clinical task.

We examined students’ strategic thinking relative to their preparation or planning before beginning the clinical reasoning task, during the actual task, and after they completed the task. In general, we found that although the quality of students’ strategic thinking may not decline immediately after receiving negative corrective feedback, it does begin to shift towards less strategic or process-oriented thinking during their second attempt to arrive at the correct diagnosis and upon receiving the second round of negative feedback. Our descriptive analysis of participant responses to the strategic planning and metacognitive monitoring questions clearly illustrated the nature of this qualitative shift in the participants’ strategic thinking. For example, following the second instance of negative corrective feedback (Time 3), approximately 50 % of the sample reported no plans or ineffective plans as they reflected on how they would attempt another iteration of the activity. In fact, only 20 % of the participants referenced specific aspects of the diagnostic reasoning processes at Time 3 when asked about their strategic plans, whereas 50 % of the participants referenced these processes in prior iterations of the task (see Table 1). The fact that students abandoned task-specific strategic thinking so quickly during the diagnostic reasoning task is compelling because the participants had recently received several months of training on this process in the same year that the study was conducted.

The declines in strategic thinking paralleled the large drops in participant self-efficacy during the task. From a theoretical perspective, the observed declines in self-efficacy are not surprising and are consistent with Bandura’s (1986, 1997) postulations that self-efficacy beliefs are most influenced by the quality of one’s mastery experiences. That is, when students struggle to perform well on a given task, particularly those who are novices or those who do not have extensive an experience with that task, their self-efficacy will often decline (Artino 2012). What was particularly striking in this study, however, was the immediacy and size of the drop in self-efficacy. That is, over the course of the 30-min clinical activity, the participants’ self-efficacy dropped from a mean of approximately 53 at baseline (based on a Likert-type scale ranging from 0 to 100) to a mean of 22 after the second round of feedback. Thus, at the end of this practice session, the average participant possessed minimal confidence that they could successfully perform the task.

These findings are particularly relevant to medical education because clinical reasoning is a core competency and because the practice session used in this study represented a close approximation of a typical practice opportunity for students enrolled in the ICR course. When medical educators engage students in practice activities involving clinical skills, it is important for them to be cognizant of how quickly student motivation and thinking can change and how providing outcome-oriented feedback about poor performance can push some students toward a maladaptive path of self-doubt and potential withdrawal or disengagement (Bandura 1997; Hattie and Timperley 2007; Pajares and Urdan 2006). Recent research in medical education has shown that outcome-oriented thinking can be detrimental to performance and the efficiency of strategic behaviors during clinical tasks. For example, Brydges et al. (2009) conducted a study to compare the effects of process and outcome goals during a self-guided learning activity targeting wound-suturing skills. The authors reported that students who set outcome goals performed worse on a transfer task than students in a process goal condition and were also less efficient in their use of instructional materials to guide their own learning.

It is important, however, to consider an alternative explanation for the drop in self-efficacy observed in this study. Although the most obvious explanation for the decrease in self-efficacy was the inability of participants to generate the correct diagnosis (i.e., they did not attain mastery of this task), one could also argue that the shifts were simply a reflection of the participants’ poor self-assessment or calibration at the outset of the study (Bol et al. 2005; Eva and Regehr 2005; Kruger and Dunning, 1999). That is, the large drops in self-efficacy could have been due to the participants’ overestimates of their perceived capabilities before they began the task. There is much research to support the premise that students are often unaware of their strengths and weaknesses and will often overestimate their capabilities and skills (Davis et al. 2006; Dunning et al. 2004; Eva and Regehr 2005; Kruger and Dunning 1999). Although we do not consider this interpretation to be particularly robust in the current study because the participants’ mean baseline self-efficacy scores were fairly modest and thus likely did not represent gross distortions in perceived capability, it is important for researchers and clinicians to be mindful of how overestimates of performance at the outset of a task can mask true shifts in efficacy that may occur over time. Further, when medical students do not accurately self-assess, such as overestimating their skills or performance, during challenging or difficult case scenarios they will often not make appropriate decisions about when, how, and why changes in their strategic approaches to clinical activities are needed.

Although we believe that results of this study make an important contribution to the medical education literature, the study has some limitations. First, the participants were recruited from a single institution and were relatively homogeneous in terms of their diagnostic reasoning skills. Ascertaining whether our findings generalize to other institutions and populations across the developmental spectrum (e.g., medical students with limited applied experiences versus residents and other more experienced clinicians) may be a fruitful line of research. Along the same lines, it is important for researchers to consider how certain variables (e.g., prior achievement) may moderate the relation between types of feedback and regulatory and motivation reactions. For example, although it seems reasonable to speculate that most medical students possess strong histories of academic success and thus will persevere through minor setbacks or challenges, it is equally plausible that some medical students with a more inconsistent record of academic achievement will be quite sensitive to negative feedback and thus be more vulnerable to reacting in maladaptive ways when they do not attain immediate success on a given task.

Further, it is well known that when medical students transition to the clinical phases of training, they often fail to ask for help, a particularly important regulatory strategy (Karabenick and Berger 2013), even when they need it (Artino et al. 2012; Kennedy et al. 2009). As such, exploring how feedback might influence motivation beliefs and regulatory processes in authentic clinical settings is another line of research that may be of interest to clinical educators. Moreover, given the recent work showing that medical students prefer certain types of task feedback than others (i.e., feedback that facilitates normative comparisons rather than self-comparisons; Manzone et al. 2014) even though such feedback often undercuts student motivation and regulation, much more research is needed to explore the interplay among types of feedback, student perceptions and processes, and clinical performance outcomes.

Another limitation of this study was that we only provided a single type of feedback to the participants. Future research should use SRL microanalytic protocols and/or other contextualized forms of SRL assessment to examine the differential effects of feedback type (e.g., corrective versus process) on the motivation and self-regulatory processes of medical students as well as the stability of students’ regulatory responses over time or across other clinical tasks. It may also be beneficial to employ experimental controls to examine the causal links between feedback and shifts in SRL. Finally, we did not investigate how the participants interpreted the feedback that was provided to them. Examining how individuals interpret different types of feedback may be a critical factor in understanding how and why they adapt or strategically engage when struggling to perform a clinical task (Eva et al. 2012).

## Conclusion

In this study, we showed how SRL microanalytic protocols could be administered as part of a clinical reasoning activity to document shifts in medical students’ motivation and regulatory processes during that activity. We found that when novice learners are not immediately successful on a diagnostic reasoning task, their self-efficacy plummets and they struggle to sustain their strategic engagement during the activity. Although it is clear that much more research needs to be done regarding the links between feedback and the patterns of SRL processes exhibited by medical students, it is important for medical educators to be cognizant of the dynamic and fluid nature of SRL. Further, future research should begin to examine the acceptability and utility of contextualized forms of SRL assessment, such as SRL microanalysis, for improving the quality with which medical educators provide instruction, remediation, and other clinical experiences for students.
